# Oral tolerance, a potential driver of systemic disease risk in periodontal patients

**DOI:** 10.3389/fdmed.2025.1700937

**Published:** 2025-12-16

**Authors:** Alan E. Hubbs

**Affiliations:** Rare Opportunities LLC, Rockville, MD, United States

**Keywords:** oral tolerance, periodontitis, cardiovascular disease, chronic inflammatory disease, GALT, immune switching, infective endocarditis, gut-associated lymphoid tissue

## Abstract

Periodontal disease is a recognized risk factor for sepsis, infective endocarditis, and cardiovascular disease. Oral bacteria, including viridans group of Streptococci, abundant in healthy oral microbiota, are frequently implicated in bacteremia and chronic inflammation, both contributing factors to systemic diseases. Yet the immunological mechanisms linking oral and systemic disease remain incompletely defined. This article proposes that “oral tolerance”—the immune system's suppression of responses to antigens repeatedly encountered via the oral route—may heighten susceptibility to systemic diseases, by impairing early systemic innate immune engagement with oral bacteria entering the bloodstream. Specifically, tolerogenic-signaling by gut-associated lymphoid tissue may condition circulating innate immune cells toward a regulatory phenotype, delaying effective pathogen clearance and promoting cytokine disorientation. This brief review outlines mechanistic insights, explores microbial influences, and suggests experimental approaches, aiming to advance research in oral-systemic health.

## Introduction

The association of oral bacteria with systemic disease has been known for decades (1), and periodontal pathogens have been statistically connected to a wide variety of diseases (2, 3). Oral bacteria are known to transmigrate to the bloodstream through the gingiva—following routine dental procedures, or even as a result of ordinary chewing or brushing (4, 5)—likely facilitated by the close anatomical proximity of the subgingival space to the gingival vascular network (6). Odontogenic bacteremia is a common physiological event that is typically subclinical and transient (7), as the vast bulk of invading pathogens are cleared from the blood in under an hour—primarily through the actions of the reticuloendothelial system (4, 6, 8, 9)—followed by an efficient innate immune engagement targeting any persisting pathogens. However, in some cases, bacteremia progresses to sepsis (6, 10) or other systemic diseases.

Poor dental health leading to gingival degradation progresses to periodontitis and increases the frequency of bacteremia (1, 7). Periodontal pockets act as a reservoir of pathogenic oral bacteria resulting in high bacterial loads (1, 7) and accounting for the detection of such bacteria in diseased vascular tissues (11, 12). However, while periodontitis facilitates bacterial entry into the bloodstream, the presence of oral bacteria in cardiovascular tissues should not be conflated with direct or sole causality of cardiovascular disease (CVD). Likewise, the parallel progression of chronic periodontitis and CVDs are not necessarily causally connected. Additional studies and a detailed understanding of the mechanisms that promote chronic systemic diseases consequent to periodontitis is of primary importance (11, 13).

“Oral tolerance”—a phenomena recognized since the early 20th century (14, 15)—promotes the tempering of systemic immune responses against specific antigens through their repeated oral ingestion (16, 17). This report provides evidence that the link between periodontitis and systemic disease may involve gut-associated lymphoid tissue (GALT), the primary site of oral tolerance induction. In support of this hypothesis, an exploratory survey of the literature—focused on infective endocarditis (IE) as a representative systemic disease—is presented, and a plausible mechanism is described.

Oral bacterial strains are routinely detected in association with IE. In a 200 patient study, within the Group B cohort—40 confirmed cases of IE (17 with and 23 without periodontitis)—the most predominant blood culture isolates recovered from both the blood and oral cavity were viridans group streptococci (VGS), followed by *Staphylococcus aureus,* and thereafter *Enterococcus spp.* (18). As a group, HACEK bacteria—the predominant upper respiratory tract (URT) resident found in infective endocarditis—are also found in the mouth and are proposed to enter the blood through the gingiva (19). Beyond endocarditis, some oral bacterial strains have been repeatedly detected in association with chronic cardiovascular conditions, suggesting not only immune evasion but durable systemic residency (7, 13, 20). As examples, *Porphyromonas gingivalis, Tannerella forsythia*, and *Campylobacter* are associated with chronic cardiovascular disorders such as atherosclerosis (20), and in animal experiments, infection with *P. gingivalis* led to atherosclerotic changes and systemic inflammation (21).

Mounting evidence points to inflammatory mechanisms as the primary link between periodontitis and cardiovascular disease (22, 23). Chronic periodontitis occurs in a context of a pathogenic shift in the subgingival microbiome—with induction of an innate immune response—and drives exacerbated and dysregulated inflammatory responses associated with atherosclerosis, hypertension, thrombosis, and stroke (11). As such, there is evidence of that chronic periodontitis promotes exaggerated systemic inflammatory responses known to directly contribute to cardiovascular and other systemic diseases. Taken together, a framework describing chronic inflammation associated with periodontal disease progression, and inflammatory cytokine profiles—mediated by immune dysbiosis—has been proposed as primary contributor to both local and systemic disorders (21).

In this classic model, chronic periodontitis supports repeated bacterial transmigration to the blood leading to systemic immune engagement and driving “exacerbated and dysregulated inflammatory responses.” Chronic local periodontal inflammation is followed by chronic systemic inflammation and each drives a cycle of continued progression of disease, locally and systemically. This explanation is logically consistent, supported by strong evidence implicating chronic inflammation, and is widely accepted. Admittedly however, “immune, inflammatory, and systemic features of periodontitis and its many related diseases are far from being fully understood and are indeed still debated” (21).

We suggest this model simply remains incomplete, not accounting for all aspects of disease attribution, or the precise mechanisms underlying the dysbiotic immune state. Within this canonical model, the transmigration of pathogens into the bloodstream—followed by innate immune detection—is considered the initiating event. However, this view is limited as it focuses exclusively on systemic immunogenic engagements within the blood. This implicitly assumes that prior mucosal encounters with the same pathogen are also uniformly immunogenic, and that any resulting immune memory decays passively over time. It disregards the plasticity of immune posture through active immune reprogramming or tolerance induction. The failure to consider how immune states may be reshaped, suppressed, or re-tolerized in the course of repeated mucosal and systemic immune engagements leaves a gap in the causal chain. We propose that immune tolerance, as shaped by repeated tolerogenic mucosal exposure via the oral route—“oral tolerance”—offers a mechanistic explanation for immune dysbiosis and its systemic consequences within the inflammatory model.

Immune reactions may be immunogenic or tolerogenic in nature and they may be conveyed systemically throughout the body or they may be compartmentalized locally. Mucosal associated lymphoid tissue (MALT) sites—where antigen-specific tolerogenic or immunogenic responses are moderated in part through regulatory T cells—are considered integral in balancing immunity and tolerance at portals of antigen entry to the body, such as the oral cavity, respiratory tract, gastrointestinal tract, and urogenital tract (24). These sites of organized mucosa (i.e., Peyer's patches, tonsils, and mesenteric lymph nodes, etc.) are composed of specialized lymphoid follicles that differ in their cellular composition and antigen sampling mechanisms. GALT—the largest mass of immune lymphoid tissue in the body—is a highly specialized subtype of MALT that is located in the small intestine.

Generally, innate immune engagements at MALT sites, result in responses that are confined to the specific anatomical compartment from which the immunogen is sampled (24). The oral cavity, for example, exhibits a local baseline level of tolerance to resident bacteria which is mediated by MALT (25), promoting immune homeostasis and the controlled immune response observed in association with gingivitis prior to the advancement of periodontitis. This balance is achieved when gingival mucosal tissue engages in immunogenic signaling—via dendritic cells, T cells, and neutrophils—while at the same time organized MALT structures surrounding the tonsils mediate local tolerogenic responses (25). Eventually, as periodontitis progresses this dynamic equilibrium within the oral cavity is overcome, likely through local inflammatory cytokines such as IL-17 (26), associated with dysbiosis of the oral microbiota (27). In humans, Th17 cell defects have been associated with diminished periodontal inflammation and bone loss, implicating Th17 in the local inflammatory processes; and in a mouse model, experimental introduction of dysbiotic changes in the oral microbiota led to elevation of Th17 cells—and their inflammatory cytokines—contributing to local inflammation (28). Although the oral mucosa is not particularly noted for the direct conveyance of immune responses to the systemic arm of the immune system—these processes may potentially exert secondarily immune effects systemically through diffusion of inflammatory cytokines (7, 29) to the circulation or adaptive B-cell antibody production.

GALT responses have greater reach than other mucosal immune responses as they may be tolerogenic and systemic or may be immunogenic and compartmentalized. For convenience, Table 1 summarizes GALT-mediated responses as a function of antigenic inputs. The right most column of the Table describes the response when Pattern Recognition Receptors (PRRs) on innate cells bind Pathogen-Associated Molecular Patterns (PAMPs)—present on live pathogens or fragments thereof—initiating an immunogenic signaling cascade much like systemic immune responses (30, 31). GALT then mediates activation of T and B cells, which are programmed to recirculate and seed distal sites of the intestinal mucosa resulting in compartmentalization to the gut (32). However, depending upon the prevailing cytokine milieu, GALT may alternatively promote systemic priming or inflammation.

**Table 1:** GALT antigenic stimuli and immunological responses.

Immunological aspect	“Soluble antigens”	“PAMP bound antigens” (whole bacteria and fragments thereof)
Antigenic stimulus	Soluble antigenic epitopes released from swallowed food or bacteria	Whole bacteria and bacterial fragments that display PAMPs (e.g., LPS, flagellin)
Sampling mechanism	M cells, dendritic cells (DCs) in Peyer's patches	M cell, DC, and epithelial PRRs (e.g., TLRs, NLRs) through binding of PAMPs
Naïve CD4⁺ T cell differentiation	Treg induction (expressing FoxP3+, IL-10+, TGF-*β*+)	IL-12 → Th1 induction IL-6 + TGF-*β* + IL-23 → Th17 induction
Cytokine release	TGF-β, IL-10 (anti-inflammatory, tolerogenic)	IL-6, IL-1β, IL-12, IL-23 (pro-inflammatory, immunogenic)
B cell responses	Local quiescence through IgA class switching (i.e., low-affinity T Cell independent) and IL-10 release	Immunogenic through T Cell-dependent IgA and IgG production, (i.e., higher affinity, inflammation-linked)
Net GALT generated response	Systemic tolerogenic via Treg migration to the circulation and peripheral tissues	Local immunogenic with neutrophil recruitment, and barrier modulation, via effector T Cells homing back to the gut
Immunological outcomes	Systemic tolerance with or without local immune quiescence via homing back of regulatory B-cells to the gut.	Compartmentalized inflammation with or without potential systemic priming depending upon the antigenic and cytokine context
Physiological outcomes	Supports commensal stability, moderates immune posture	Gut protection against bacterial seeding, but chronic or excessive exposure may contribute to microbiota dysbiosis

Mixed exposure to soluble antigens, PAMP-bound antigen, and live bacteria, likely evokes a nuanced balance of tolerogenic and immunogenic signaling shaped by dose, humoral and cytokine context, and microbial viability.

GALT conveyance of a tolerogenic posture to the systemic immune system—via direct cellular interactions and cytokine signaling—depends upon antigen engagement in the proper cytokine context (16, 33). The middle column of Table 1 describes the response of GALT upon processing soluble antigen from swallowed food or bacteria. Ordinary digestive processes of the stomach release soluble unbound antigens likely creating a mixture of free antigens and bacterial fragments. Specialized subsets of innate dendritic cells (DCs)—located in the GALT adjacent functionally-integrated lamina propria—sample these luminal antigens and migrate to the mesenteric lymph nodes where they present antigen, in a non-inflammatory cytokine context inducing naïve CD4⁺ T cells to differentiate into Tregs. Once differentiated, these induced Tregs migrate back to the lamina propria, guiding immune tolerance and tempering inflammatory responses through the release of cytokines such as IL-10 and TGF-*β* (34). In parallel, macrophages conduct bacterial clearance and secrete IL-10 (24). Studies show that IL-10 and TGF-*β* act synergistically to suppress B cell activation—highlighting how cross-talk between systemic and mucosal immunity interact to establish and reshaped immune posture (24, 35), potentially toward tolerance, as in this example.

The direct conveyance of tolerance to the systemic innate immune system by GALT—in response to swallowed antigens—is essential for: preventing inappropriate immune responses to ordinary dietary antigens, maintaining symbiosis with a vast array of commensal bacteria, (34, 36), and preventing autoimmunity (17, 34). Further, the noninvasive therapeutic induction of oral tolerance as a means to achieve systemic immune modification—including the treatment of autoimmune disease (16, 33, 37, 38)—demonstrates the plasticity of the innate response and a reprogramming of systemic immunity based upon environmental cues. These facts alone provide the basis for consideration of oral tolerance within the context of the inflammatory model. Moreover, if access to GALT by oral bacteria is shown to be associated with risk of systemic disease, it will suggest a role for oral tolerance in disease development.

While oral tolerance has not previously been implicated in sepsis or endocarditis, this report explores a novel hypothesis: that GALT, functioning as designed—through the induction of oral tolerance—may contribute to the observed linkage between periodontitis and cardiovascular disease. To our knowledge, this is the first report to suggest that oral tolerization—though mechanistically intact—may consequentially facilitate systemic disease progression.

## Methods and results

### Literature survey

We performed an exploratory hypothesis-generating analysis examining the literature using Google Scholar to determine whether GALT access is strongly associated with CVD. Bacterial strains sorted to one of five categories based upon location of primary residence were examined for association with cardiovascular disease. For each strain the number of reports of a clinical association was counted and each strain was parsed into a cardiovascular disease association category based upon whether or not the tally exceeded an arbitrarily designated threshold number of published reports. This method, of data chopping then counting, amplifies the visibility of minority disease associations otherwise obscured by the disproportionate representation of a narrow subset of bacterial strains—or taxa, such as the viridans group—in published reports. In the present case, the axis formed between location of residence and disease attribution creates a “binary attribution profile” describing the relationship between strain location and disease attribution. Foundational validation of this method hinges upon the appropriate designation of primary residence categories, such that two of the five categories represent presumptive negative and positive controls, to confirm the well-established association between periodontal strains and CVD.

### Survey method

Ninety-one bacterial strains were compiled based upon the primary consideration of having been reported to enter the blood under some conditions to cause a bacteremia. Whether the strain has been categorized as a commensal or opportunistic pathogen was ignored, as this distinction is somewhat arbitrarily defined and always evolving. Care was taken not to over or under represent any categories, but inclusion on the list was not rigorously monitored and as a practical matter, only a fraction of the documented oral bacterial strains are represented in the analysis. Bacterial strains of primary anatomical residency in the oral cavity, the mucosal surfaces of the lumen—such as the URT and the gastrointestinal tract—the skin, and the environment (provided they are known to cause infection upon entry to the blood or systemic compartment), were included.

Given our interest in exploring the potential role of oral tolerance in systemic disease associations, we faced a notable gap in the literature: no direct evidence exists regarding whether specific oral or opportunistic pathogens are subject to oral tolerization. In response, we constructed an assumption-based classification framework grounded in two key premises regarding anatomical access to GALT, which we treated as conceptual stand-ins for tolerization potential.

The first premise posits that oral strains have GALT access via swallowing. The second extends this access to strains residing in the nares, pharynx, and upper respiratory tract (URT), based on mucociliary drainage and ingestion via swallowing. The notion that URT bacteria—including soluble, particulate-associated, and whole bacteria-associated antigens—have GALT access is supported by the appearance of these strains in the gut after experimental swallowing (39, 40). Gastrointestinal strains were also considered to have GALT access, due to direct proximity. We did not specifically find reports of skin-associated or environmental strains accessing GALT and classified these as lacking GALT access in our survey. This recursive reasoning framework supports a determination of whether the survey findings—specifically, the distribution of cardiovascular disease associations across these anatomical categories—aligns with the proposed GALT access logic.

Initially, examination of literature documenting connections between specific bacterial strains and cardiovascular disease proved to be difficult, because categorization of diverse acute and chronic conditions affecting different anatomical structures of the cardiovascular system (atherosclerotic, coronary, cerebrovascular, and peripheral vascular disease, valvular heart disease, heart failure and cardiomyopathies, arrhythmias, infective endocarditis, and hypertension) all fall into the category of CVD in the literature. Therefore, a more focused and clearly defined examination of strains clinically detected in association with the acute and well-defined condition of IE was selected for initial analysis, then the secondary well-defined condition of “found in atherosclerotic plaques” was applied to strains not found in association with IE. Together, these criteria were considered sufficient to represent the broader CVD spectrum and the results do not consider whether strains are associated with both conditions.

The analysis was conducted manually using Google Scholar. A more robust literature examination is reasonable before a true statistical analysis would be able to be applied, as this sole source examination risks some level of bias. The use of Google Scholar was an expedient compromise as it scrapes from many publisher sites, repositories, and multiple databases providing a breadth of examination, while at the same time it avoided the need to apply deduplication protocols, as would be necessary if multiple databases—specializing in case reports—were searched, whether or not peer reviewed literature databases are included.

Database queries of the form: {*[strain name] AND (endocarditis OR cardiovascular)*} were applied. This broad filter was chosen to maximize inclusivity, with results evaluated through manual browsing to assess both the quantity and quality of reports linking each bacterial strain to IE. For strains not found to be associated with IE, a secondary query—{*[strain name] AND “atherosclerotic plaques”*}—was applied. Due to the relative ease with which the relevant references are called from Google Scholar, no references are provided for the strains in the table. The combined queries were conducted across May 2025.

The binary attribution profiling method was applied such that individual strains were assigned a binary score of **1** (one) if the search yielded either six or more case reports within the past decade, or at least one peer-reviewed article asserting association with IE or atherosclerotic plaques; otherwise, a **0** (zero) score was assigned. This analytical approach is designed to assess the influence of anatomical location—used here as a surrogate for GALT access—without measuring epidemiological burden. It treats strains equally, independent of strain frequency, severity, or abundance, by assigning equal weight to both minority and majority strains. By mapping strain-specific associations with cardiovascular disease in this manner, the analysis looks beyond features unique to the oral cavity (such as gingival transmigration). This strategy is particularly useful for identifying potential causal factors that may influence systemic disease trajectories.

### Survey results

**Figure 1:**
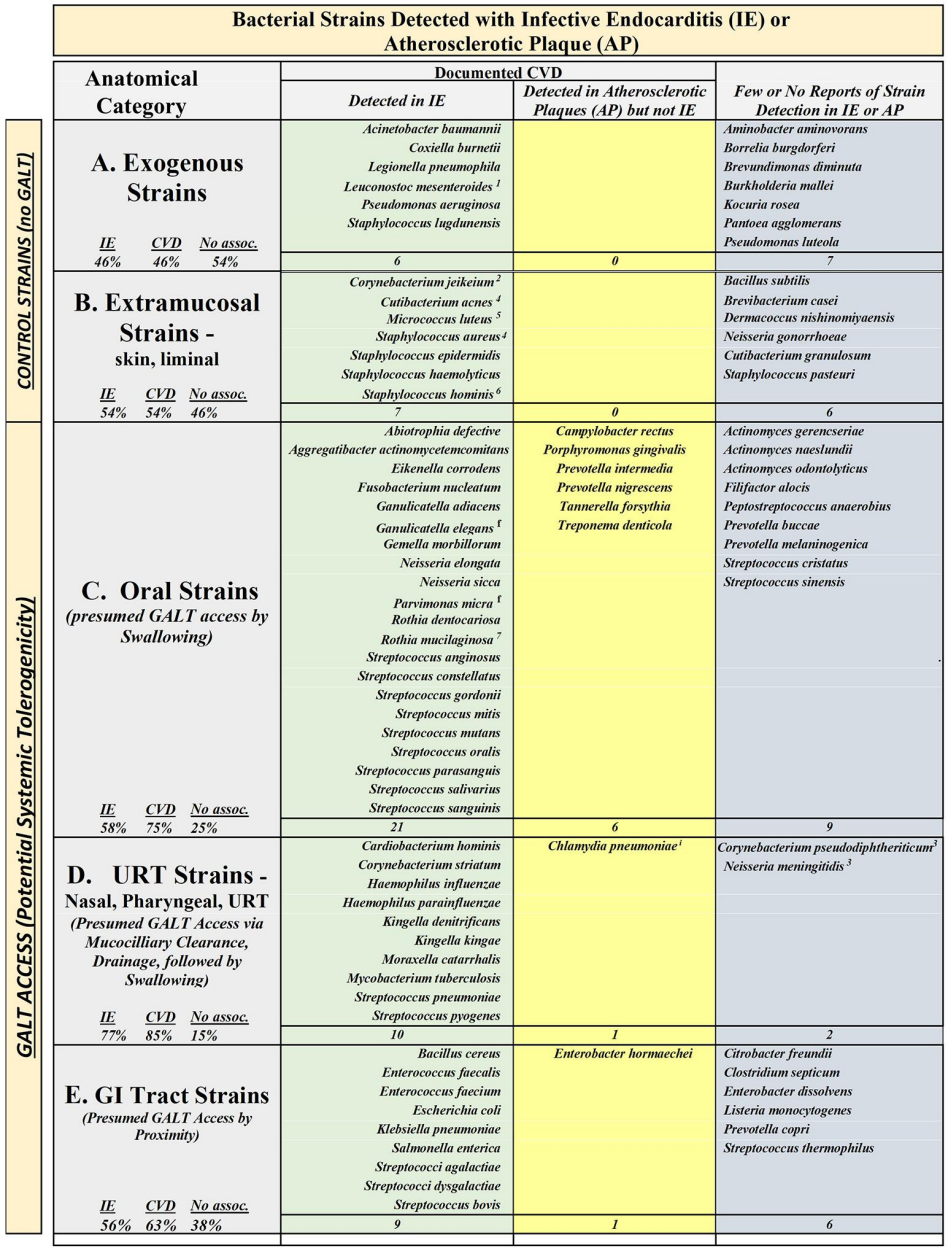
Infective endocarditis correlates strongly with GALT access. The ninety-one bacterial strains as listed in Table 1 were sorted into five categories (named in each row in the left-most column) according to their location of primary residence: Category A—13 “Exogenous”, environmental strains; Category B—13 “Extramucosal” strains; Category C—36 “Oral” strains; Category D—13 “URT” strains—including nares, pharynx (oro-, naso-, pharynx), or upper respiratory tract; and, Category E—16 “GI” (gastrointestinal tract) strains. The strain names were placed in one of three columns based upon their binary score: a score of 1 for infective endocarditis (IE) was placed in the “IE” column; a score of 0 for IE with a secondary score of 1 for atherosclerotic plaques (AP) was placed in the “AP but not IE” column; and, a 0-score for both IE and AP was placed in “Few or no reports” column. Tallies for each box of the matrix are at the bottom of each box; Percentages of IE, CVD (represented by combined tally of IE plus AP), and No Association are shown at the bottom of each box in the title column. 1. Foodborne, moderate IE, slow grower with theoretical transient GALT contact. 2. Modest number of IE case reports, potential mucosal access via border zones (axillae, groin, perineum). 3. Marginal number of case reports. 4. Liminal mucosal access pilosebaceous units, eyelids, ears. 5. Multiple Sites of Residency—Placed in Skin as the primary, also found ubiquitously in soil dust air water, mouth, mucosal surfaces, throat and upper respiratory tract. 6. Moderate number of case reports, potential border zones access (eyelids, ears). 7. Moderate number of case reports and peer reviewed article; immune compromised hosts favored. “f”—renamed or reclassified: Ganulicatella elegans formerly Abiotrophia elegans Parvimonas micra formerly Peptostreptococcus micros. “i” intracellular potential. “X” Excluded from analysis based on complexity of characteristics, circumstances, or rapid lethality: Helicobacter pylori, Yesrsinia pestis.

Supplementary Table 2 containing the complete list of 91 bacterial strains is placed in the Supplementary Materials for reference. Figure 1 is a visual mapping of the list in the form of a matrix displaying the relationship of the anatomical site of origin (rows) vs. disease attribution score (columns) represented by the relevant strain names.

The Figure 1 strains were categorized into five categories (listed for each row, in the left-most column) according to their location of primary residence (one environmental and four anatomical)—each defining a row in the chart: Category A—13 “Exogenous”, environmental strains; Category B—13 “Extramucosal” strains; Category C—36 “Oral” strains; Category D—13 “URT” strains—found in the nares, pharynx (oro-, naso-, pharynx), or upper respiratory tract; and, Category E—16 gastrointestinal tract (GI) strains.

Each strain was analyzed and assigned to one of three columns in the matrix according to their attribution: to IE (*infective endocarditis*); to AP (*atherosclerotic plaques*); or, to neither EI or AP—in accordance with the binary scoring method as described above. The combined total of the attributions for each column were 55 strains attributed to—*having reports documenting detection of the strain in association with*—IE, 8 strains attributed to AP—*after having been found to not have attribution to IE*, and 28 strains with no significant attribution to either IE or AP. For clarity, the analysis did not assess whether strains may have a dual association with both IE and AP.

The presentation is augmented with a tally of the number of strains falling within each matrix element, and the percentages of the tallies as a portion of the total entries for each row are beneath the row titles (to the left side of each row).

Figure 1, Row A, Exogenous Strains had attribution scorings of 46% to IE; 0% to AP alone; and, 54% to neither IE nor AP (no CVD attribution).

Figure 1, Row B, Extramucosal Strains had attribution scorings of 54% with IE; 0% with AP alone; and, 46% neither IE nor AP.

Combined or taken separately, the Row A and Row B represents an approximate even distribution of strains attributed to CVD as compared to strains not attributed to CVD—strains were neither over-represented nor under-represented in regard to their attribution to CVD. As such, the Exogenous and Extramucosal groupings represent a reasonable control set of strains that do not access GALT (*GALT-negative control set*), for comparing strains of different anatomical locations in regard to GALT access and CVD attribution.

Figure 1, Row C, Oral Strains had attribution scorings of 58% with IE; 17% with AP alone; and, 25% neither IE nor AP. Within the oral strain category, this represents a 2.3-fold over-representation of attributions to IE compared to strains not attributed to IE; and 3-fold over-representation for attribution to CVD compared to not attributed to CVD—indicating that a large portion of strains of the oral cavity have been found in association with CVD. More importantly, a 1.5 fold-greater portion of oral strains are reported as found in association with CVD as compared to the non-GALT stains (Categories A plus B).

The proportion of oral strains attributed to CVD, relative to the GALT-negative control set, affirms the significance of oral strains as contributors to cardiovascular disease. Simultaneously, the comparison between CVD-attributed strains and unattributed strains, within the overall pool of oral strains, reveals that a wide spectrum of oral strains have been found in association with CVD. The strain-level tally demonstrates that systemic relevance within the oral strain set extends beyond the narrow list of traditionally aggressive or periodontitis-associated strains—revealing a broader capacity among commensals to promote disease than might be inferred from the disproportionate focus in published literature on opportunistic pathogens linked to periodontitis, infective endocarditis, and CVD.

Figure 1, Row D, URT Strains—including the nares, sinuses, pharynx, oropharynx, nasopharynx, and upper respiratory tract—had attribution scorings of 77% with IE; 8% with AP alone; and, 15% neither IE nor AP. This over-representation of URT strains in CVD-associated contexts suggests that URT-resident strains may share, with oral strains, a comparable systemic potential to promote cardiovascular disease. Based upon our key assumption regarding alimentary access conferring GALT engagement, strains from these regions are interpreted as having systemic immune relevance. These findings support the anatomical logic of GALT-mediated linkage between peripherally located strains and systemic disease.

Figure 1, Row E, GI Strains—gastrointestinal residents presumed to have GALT access due to anatomical proximity—had attribution scorings of 56% with IE; 7% with AP alone; and, 38% neither IE nor AP. This pattern aligns with the broader survey trend, with 1.66-fold more strains from the GI that are able to contribute to CVD than unassociated GI strains.

Taken together, the anatomical distribution of IE- and CVD-associated strains is in alignment across all sites with access to GALT. In contrast, exogenous and extramucosal sites—those lacking GALT access—exhibit a markedly weaker attribution profile toward IE. Notably, strains from “non-oral” GALT-accessible sites mirror the attribution patterns of oral strains. The results reflect a link between oral strains and IE, consistent with the well-established connection between periodontitis and CVD. The results also reflect that IE linkage extends beyond oral strains to include—sites that have access to GALT via swallowing—an anatomical characteristic in common with the oral cavity.

The survey was not designed to detect whether GALT-accessible strains share strain-specific physical traits such as endothelial adhesion molecules (e.g., surface proteins) and immune evasion mechanisms (e.g., biofilm formation) which may facilitate CVD association. The results show that GALT access may function as an environmental enabler—broadening the pool of strains eligible for endocardial attribution. Binary analysis of strain association with CVD confirms that oral pathogens do not hold exclusive rights to the pathogenesis of IE. While they dominate clinical relevance—by virtue of reported transmigration frequency, case prevalence, and literature saturation—the comparable proportion of IE-associated strains from non-oral origins reveals a deeper truth: causality is not a periodontal privilege. Instead, a shared feature across anatomical origins emerges: access to GALT, with direct immunological implications.

## Discussion

Oral opportunistic pathogens are at the axis of the disease link between periodontitis and CVD. As such, oral tolerance—mediated by GALT, primarily in response to swallowed soluble antigens—is the immune tolerization mechanism central to our considerations. In order to evaluate whether or not inclusion of oral toleration within the inflammatory model is warranted, a rudimentary understanding of immunogenic and tolerogenic immune mechanisms is necessary—and will be assumed herein. The reader is encouraged to consult the following helpful sources covering the complex cascade of cytokine signaling and its ensuing effects (41, 42, 43), and the mechanisms and prospects for oral tolerance (16, 17, 33, 37, 38).

### Proposed causal mechanisms

The proposal that oral tolerance plausibly contributes to systemic disease is logically based in the temporal consequences of “Immune Switching”, a process by which an existing immune posture is reprogrammed in response to an invading pathogen or other stimuli. In the present hypothesis, an existing predisposition to tolerance will be reflected in the phenotype of circulating innate immune cells—and a prevailing tolerogenic cytokine milieu—a state fundamentally antithetical to the immunogenic posture required for timely redress against an invading strain. Time is required to upregulate gene expression related to intracellular signaling, cell surface proteins, and cytokines, in order for innate cells to switch to a ready state phenotype prepared to conduct an efficient immunogenic response against a pathogen to which the immune system has been tolerized. These necessary changes of gene expression and protein surface display may take hours depending upon the prevailing intracellular and extracellular conditions, thereby disturbing the efficient rollout of immune engagement.

**Figure 2:**
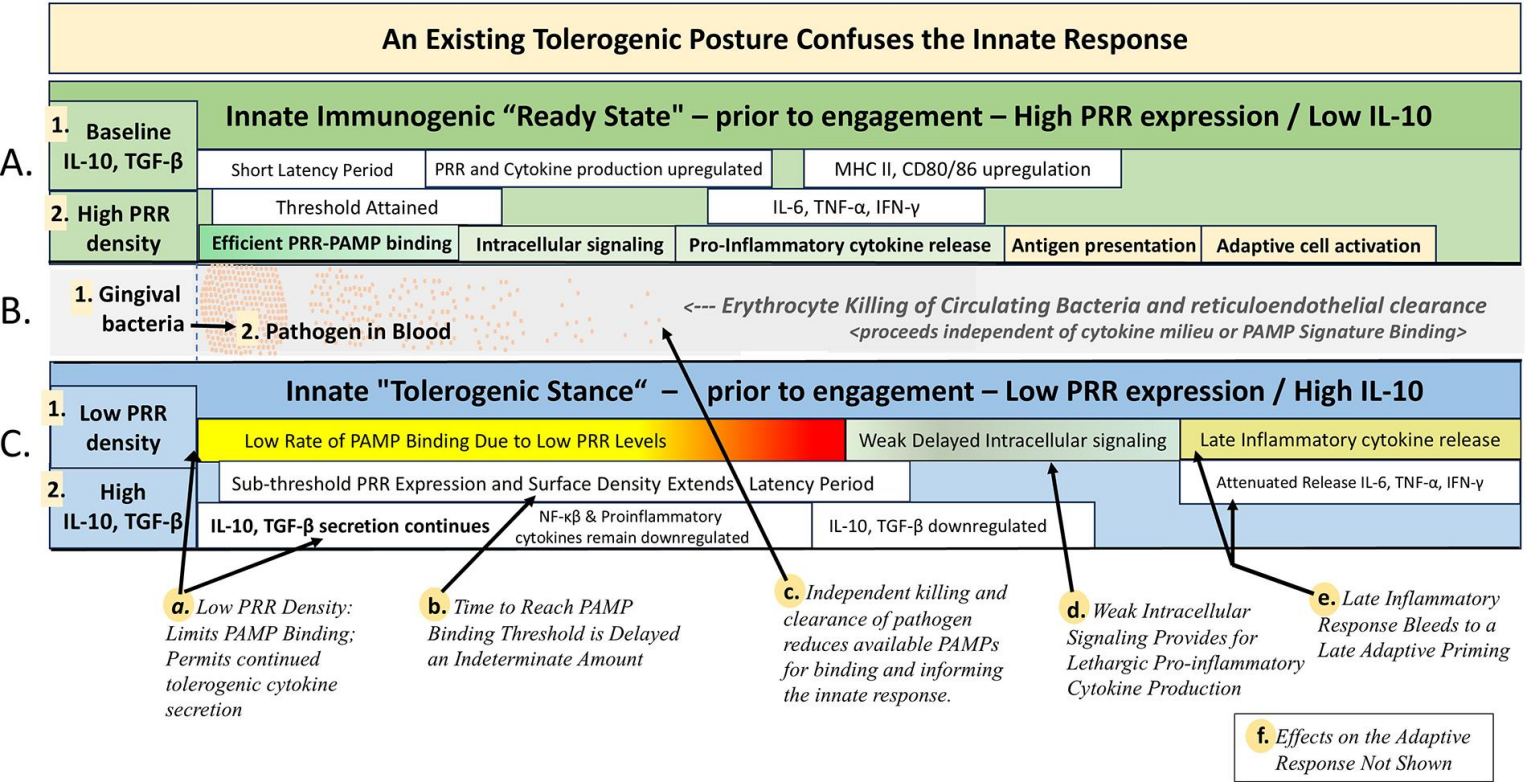
Effects of a preestablished tolerogenic posture on systemic innate immune engagement. **Part A** (green background) illustrates the progression of systemic innate immune activation from a pre-existing naïve or immunogenic baseline—termed the *Immunogenic Ready State*. Baseline cytokine levels and pattern recognition receptor (PRR) density on resident innate immune cells are depicted at the left. Mechanistic steps are represented by sequential boxes aligned left to right, with box length representing the temporal duration of each phase. **Part B** depicts the independent action of the reticuloendothelial system in clearing circulating bacteria, operating outside the influence of innate immune cell activity. **Part C** (light blue background) presents a parallel progression of systemic innate engagement originating from a pre-established *Tolerogenic Posture*. This pathway is vertically aligned with **Part A** to permit direct comparison. Baseline cytokine levels and PRR expression on innate cells are again shown at the left, with mechanistic steps sequenced left to right and box lengths reflecting estimated time intervals. Callouts “a” through “f” annotate key differential effects observed during immune switching from a tolerogenic to an immunogenic state.

The plausibility of this model rests on the contention that at an early age, prior to periodontitis, individuals swallow low doses of oral bacteria routinely over the course of years—providing for GALT sampling of soluble proteins and bacterial fragments of these bacterial strains—in a manner that is consistent with the requisites for GALT mediated tolerogenic signaling to the systemic innate immune system. Repeated antigen-specific low-dose exposure is generally associated with durable oral tolerance—particularly through the induction of antigen-specific inducible Tregs—while high-dose exposure can also induce tolerance, but often through deletion or anergy, which may be less durable (44). The mechanistic consequences of a single instance of Immune Switching are summarized visually in Figure 2.

### A model of innate engagement in the context of an existing tolerogenic posture

The Chart depicts the consequences of a hypothetical example of Immune Switching, by juxtaposing the ordinary time progression of the innate immunogenic response—from a naïve immunogenic “ready state” (Figure 2, Parts A-1 and A-2)—with the ordinary course of reticuloendothelial clearance of pathogen from the circulation (Part B), and a hypothetical alternate timeline in which the innate response unfolds within the context of a pre-existing tolerogenic immune posture (Parts C-1 and C-2).

In the immune ready state (A), cytokine expression is at a low baseline level (A-1), and the population of circulating innate cells (dendritic cells and macrophage) display a phenotype of high PRR density on their surface (A-2). This promotes an efficient innate engagement upon introduction of a pathogen into the bloodstream. Vigorous PRR binding of PAMPs occurs immediately upon pathogenic entry into the circulation and threshold levels are reached and trigger intracellular signaling with a minimized period of latency. As a result, pro-inflammatory cytokine release (IL-6, TNF-α) is timely and leads to early cell to cell antigen presentation and efficient adaptive cell activation.

The depicted role out of the innate immunogenic response represents a set of coordinated and well-timed interactions and release of inflammatory cytokines from systemic innate cells. These cytokines are necessary for activation of the adaptive immune system, which responds immediately but typically reaches full engagement only after multiple days (43, 45). In contrast, reticuloendothelial clearance of pathogen from the bloodstream, as depicted in Figure 2, Part B, is initiated—immediately upon pathogenic entry—in parallel with and independent of the innate response. This clearance process includes erythrocyte killing and reticular clearance of killed pathogen. Importantly, it does not rely upon cytokines or other aspects of the innate response—and is critical to bacterial clearance from the blood—but in the context of a delayed innate response it may exert potentially deleterious indirect effects upon the efficiency of the innate engagement.

The tolerogenic posture (Figure 2, Part C), unlike the ready-state posture, is characterized by a low density of PRR on their surface (C-1), as a consequence of downregulated expression. Additionally, anti-inflammatory cytokine (IL-10, TGF-*β*) expression is high (C-2) and such a cytokine balance promotes maintenance of a tolerogenic immune posture. The consequence of tolerogenic immune priming is a context in which the population of circulating innate cells are not prepared for an aggressive innate response, and as a result, orchestration of all of the downstream processes of innate engagement are delayed and attenuated.

Callouts (C, a—f) summarize the relevant consequences of an innate immunogenic rollout in the face of a pre-existing tolerogenic posture along with parallel pathogen clearance by the reticuloendothelial system (Part B). First, as a result of the low PRR density—PAMP binding proceeds at a comparatively lower rate—and the anti-inflammatory cytokine environment is supportive of continued low PRR expression and also continued anti-inflammatory cytokine secretion (Callout “a”). Independently, reticuloendothelial clearance (Part B) begins immediately upon invasion of the blood (B-1), and proceeds unaffected by PRR expression, PAMP binding, or cytokine secretion—such that bacterial clearance (B-2) occurs at a constant rate regardless of the immune posture—largely reducing the bacterial load from the blood within just 20 min post exposure (4), and near disappearance of detectable bacteria has been noted in under 60 min in humans (6), while mouse experiments show greater than 99% clearance in as little as 5 min (46). In the face of rapid reticuloendothelial bacterial clearance from the circulation, low pathogen concentrations may further extend the time for PAMP binding to reach threshold levels (Callouts “b” and “c”), greatly extending the latency period. Further, prior to the repopulation of “ready” innate cells—displaying a high PRR surface-density—intracellular signaling will be weak and delayed (Callout “d”)—leading to a lethargic pro-inflammatory response (callout “e”). These combined consequences may also generate conditions that affect the adaptive response as well (Callout “f”), not shown in the chart.

The events depicted in Figure 2 outline a series of sequentially accumulated mechanistic delays consequent to the Immune Switching process. While the precise magnitude of these delays is not readily predictable without direct experimental testing, extrapolations from published kinetics suggest that the cumulative latency—from PRR upregulation, to initial PAMP binding, to threshold engagement, to intracellular pathway activation—may exceed 60 or 90 min. This compares to an ordinary latency of less than 30 min.

An overview of the known mechanisms of systemic innate immune engagement is helpful to understanding the temporal disturbance of the mechanisms detailed in our model. Absent tolerization, a neutral immunogenically-ready innate phenotype is maintained by anti-inflammatory cytokines at a baseline level supporting homeostasis. Upon entry into the bloodstream, an invading pathogen is rapidly engaged by innate immune cells—including monocytes, neutrophils, and dendritic cells—via PRR binding of PAMPs, such as TLRs and NODs (toll-like receptors, and nucleotide-binding oligomerization domain-like receptors), which recognize conserved microbial PAMP signatures and initiate binding (41, 43). This engagement occurs in parallel with reticuloendothelial clearance mechanisms—operating independently of cytokine signaling and immune activation (8)—clearing circulating bacteria within minutes (4, 6, 7, 46). But, despite dramatic initial reductions in bacterial concentrations through reticuloendothelial clearance—some bacteria persists for sixty or more minutes—presumably beyond the window of detectability (6). These persisting bacteria are the critical target for immune mediated clearance.

Importantly, PAMP–PRR interactions must surpass a requisite stimulatory threshold of binding before downstream intracellular signaling is triggered—a period of latency—during which NF-*κ*B and MAPK pathways are activated but not yet transcriptionally productive. Only after this delay are pro-inflammatory cytokines—including TNF-α, IL-1β, and IL-6—expressed and released (41). These cytokines initiate the acute phase of the inflammatory cascade, drive endothelial activation, and recruit effector leukocytes—laying the groundwork for antigen presentation and adaptive immune priming (41).

Beyond the ordinary innate response as described above: our model predicts that in the event that oral tolerance has been induced, initial PAMP engagement will be impaired since the reticuloendothelial system will have likely largely depleted the circulating pathogen population from the blood—possibly having removed greater than 99% of the invading pathogens—thereby reducing the level of pathogen available to efficiently reach threshold levels of PAMP binding as necessary for innate activation. This temporal mismatch exacerbates the delay of all downstream innate and adaptive processes, including impairment of timely coordinated cytokine release, antigen presentation, and pathogen-specific tissue surveillance.

The consequences of our model have parallels in the literature. It is recognized that cytokine synthesis, secretion, and receptor expression is an orchestrated process forming a highly complex mutually regulating network of interactions which—if disrupted—may promote chronic inflammatory or immune disease (43). Also, it has been suggested that immunosuppression (e.g., via immunosuppressant therapy, neutropenia) can disturb the equilibrium that exists between the mechanisms of protective barrier functions—such as those of the reticuloendothelial and immunological systems—thereby allowing organisms to propagate and cause both acute and chronic infections with increased frequency and severity (7). Logically, interference with a timely innate response will exert downstream effects on the adaptive immune response—the full consequences of which are not speculated upon here—but one can expect that the disoriented cytokine environment and its impact on memory cell dynamics will alter the ordinary ramp-up of adaptive immunity.

If the Immune Switching model is valid, then the implications are clear: a preexisting tolerogenic innate state can incur mechanistic delays that increase the period of latency before an efficient immunogenic engagement can proceed in response to a tolerized invading pathogen. First, the tolerogenic cytokine milieu and the tolerogenic innate cell phenotype must be reprogrammed—a temporal delay. Secondly, the clearance of bacteria from the blood by the innate-independent reticuloendothelial process—may limit the available pathogen necessary for innate cells to reach threshold levels of PAMP binding and initiate the intracellular response—a misalignment that further exacerbates the increase in the period of latency. Delays of this order may be expected to have two clinically relevant consequences: expansion of the “window of opportunity” for pathogens to evade the immune system and potentially seed peripheral organs; and cytokine confusion resulting from the disturbance in the ordinary orchestration of the immunogenic response leading to inflammation.

### Clinical implications and chronic inflammation

Clinically, an expansion of the “window of opportunity” as discussed above, can potentially account for sepsis or acute seeding of peripheral tissues. But, significantly, the proposed mechanisms of oral tolerance as described in Figure 2 go further to suggest means by which chronic inflammation and immune dysbiosis may be initiated. In light of the canonical theory linking periodontitis and systemic disease via immune dysregulation and chronic inflammation, the identification of shared GALT access—when combined with the mechanistic considerations described by Figure 2—plausibility reframes the conversation: it is not merely dysbiosis, but the choreography of immune exposure—its route, timing, and frequency—that may underlie chronic systemic outcomes. To fully appreciate the potential role of oral tolerance in the context of the classic chronic inflammatory model, one must step back and consider the course of disease progression over time within the typical patient.

As discussed, prior to gingival damage the opportunity exists for GALT sampling of oral bacterial antigens to establish a baseline tolerogenic state toward some oral bacteria. But, tolerogenic GALT signaling moderates immune posture—it does not turn it on and off. As gingival damage progresses to periodontitis and opportunistic oral pathogens progress to an aggressive state in the context of a dysbiotic oral microbiota, more frequent bacteremic events, and the delivery of higher pathogen loads to the circulation may then prompt the innate cell phenotype to be immunogenic, breaking the tolerant stance. While the presentation of our model focused on mechanisms relevant to a shift from tolerogenic to immunogenic posture, it is recognized in the literature that a tolerogenic posture may be overcome by an immunogenic one (47), or conversely, an immunogenic posture can be subdued by a GALT mediated tolerogenic shift, as with oral anti-autoimmune therapy (17, 33).

It is important to consider that patients with periodontitis typically may experience intermittent periods of quiescence and activity (27). Periods of “remission” may be due to dental cleanings, antibiotics, improved hygiene, or other interventions, while periods of aggressive progression may occur due to inconsistent oral hygiene. Yet these oscillations in periodontal severity do not interrupt the continuous low dose swallowing of oral pathogens. It is theoretically possible that continued GALT sampling even after a cycle of Immune Switching—from tolerogenic to immunogenic—may contribute directly to cytokine confusion, or may allow for reversion to tolerance through continued ingestion.

If re-sampling and potential re-tolerization of previously tolerized and de-tolerized free antigens actually occurs then the groundwork for another round of Immune Switching is re-established, again favoring the induction of regulatory T cells and subtly undermining the architecture of immune memory. Over time, this recursive modulation may not only facilitate immune evasion but also may contribute to the development of chronic inflammation and immune dysbiosis—especially under conditions of age, comorbidity, or elevated microbial exposure. Moreover, the proposed phenomenon may occur but remain subclinical for years—prior to the development of periodontitis—while bacterial loads are low.

Our survey does not directly interrogate oral tolerance, nor does the model depicted in Figure 2 present mechanisms proven to contribute to the link between periodontitis and systemic disease. However, the shared anatomical feature of GALT accessibility supports its plausibility as a contributing mechanism in the observed association between periodontitis and cardiovascular disease, and the model provides a potential foundational explanation for the observed connection. Given the repeated, if not continuous, swallowing of oral bacteria, GALT is routinely exposed to the full spectrum of oral strains. This may induce immune tolerance to a subset of these organisms. In contrast, detection of oral bacteria by innate immune cells in the bloodstream prompts an immunogenic response. In light of these dual exposure routes, oral tolerance may lead to immune confusion.

While the survey results do not support a causal relationship, the attribution data do more than suggest—they compel further evaluation of oral tolerance as a significant factor in the link between periodontitis and systemic disease. Hypotheses should be articulated with sufficient detail to permit empirical testing. We believe that our hypothesis is plausible, biologically grounded, and sufficiently described to support investigation using currently available research methods.

### Experimental approaches

If validated, the present hypothesis offers a fresh perspective on the interplay between microbiology, immunology, and systemic health, particularly in the context of periodontal disease progression and its links to cardiovascular pathologies. The following courses of experimentation may be useful in disproving, validating, or extending the role of oral tolerance in chronic disease, with periodontitis as best-case model.

#### Retrospective literature analysis of Minor strain attributions

Further retrospective mining of the literature—especially with attention to minority strain attributions—may yield further insights into the systemic relevance of non-oral strains. Our present survey was limited to a single database allowing a risk of bias or potentially limiting important observations. Larger-scale surveys that explicitly include minority contributors can provide sufficient representation of strains from non-oral sites, enabling advanced filtering and contextual analysis to reduce sampling bias. Our manual methods employed binary scoring and a minimal heuristic framework based on two plausible assumptions: that oral strains are preferentially tolerized via GALT, and that alimentary canal exposure confers potential GALT access. A more robust computational framework—testing multiple assumption combinations in series and in parallel—may reveal patterns obscured by reductive filtering or categorical constraints, and allow reinterpretation of existing datasets without discarding potentially informative data.

#### Prospective clinical study design

Prospective clinical studies may be designed to include key measures capable of refuting, confirming, or refining the proposed mechanistic delays from mounting an immunogenic response under a prevailing tolerogenic posture. Future research should take advantage of the most detailed molecular biology techniques to elucidate how periodontal disease affects inflammatory and immune response pathways to affect systemic disease and to identify causal bacterial strains (48). Frequent blood sampling immediately following dental extractions may help determine whether oral tolerance modulates the systemic immune response. While timing studies have been conducted previously, newer methods may allow more frequent, less invasive blood sampling (improved resolution), Elispot-based cytokine analysis (49), and detection of minority pathogens within the bacteremic population. Comparison with control sampling immediately prior to systemic immune engagement may be able to map the time course of cytokine shifts consequent to pathogen invasion—potentially discriminating temporal elements of our model—while sampling at longer intervals may assess downstream effects on the adaptive immune system.

#### Animal model studies

Animal models offer a direct means of measuring Immune Switching effects on the latency period and testing whether antigen-induced oral tolerance increases susceptibility to bacteremia and sepsis. Murine knockout models—such as IL-10 and TLR4-deficient strains—may be particularly valuable for dissecting the roles of tolerogenic and innate pathways. Oral tolerance can be induced in mice using antigens from specific oral bacterial strains, such as Streptococcus mitis, S. oralis, or other prominent non-viridans residents of the oral cavity. Repeated oral exposure may be conducted using soluble antigens, killed bacteria, or both, with sham antigens serving as controls. Subsequent susceptibility to bacteremia and sepsis can then be assessed in a strain-specific manner.

*In vitro* cytokine profiling via Elispot may be performed at early time points to track Immune Switching dynamics. These measurements can help distinguish systemic innate responses initiated from a neutral posture vs. those reshaped from a tolerogenic baseline.

The examples provided are not intended to be exhaustive or prescriptive in terms of experimental design. Rather, they serve to illustrate that the oral tolerance hypothesis is directly testable. Results from such studies may form a foundational knowledge base for assessing therapeutic interventions, regardless of whether oral tolerance is ultimately confirmed or refuted.

## Conclusions

This paper presents an overlooked axis of connection: the role of GALT access in mediating the association between periodontitis and infective endocarditis (IE). Oral tolerance, initiated via GALT, may attenuate the efficiency of innate immune activation against oral bacteria—shaping systemic outcomes through three interlinked mechanisms. First, delayed innate activation may extend the window of opportunity during which pathogens can seed peripheral tissues. Second, disrupted cytokine timing may induce immunological confusion, prolonging inflammation. Third, repeated cycling between tolerogenic and immunogenic states may foster immune dysbiosis.

This proposed link reframes the model of interplay between microbiology, immunology, and chronic inflammation. It offers a mechanistic rationale for why some periodontal patients progress to severe outcomes—such as sepsis or cardiovascular disease—while others remain unaffected. If validated, this framework could inform therapeutic strategies, including prophylactic reversal of pathogen-specific tolerance to enhance clearance and reduce systemic risk.

Central to this hypothesis is the dual-route model of immune engagement: anatomically distinct exposures to the same pathogen that elicit opposing immune postures—tolerogenic via GALT, but inflammatory via transmigration to the bloodstream. This model does not imply a failure of oral tolerance, nor does it classify oral tolerance as pathogenic, but rather it reveals a conflict between two functional immune modes that may disrupt homeostasis under chronic or repeated exposure. Specifically, repeated exposure cycles comprised of live gingival pathogens in the circulation and soluble bacterial antigens sampled in the gut, may trigger cycles of Immune Switching—oscillations between tolerance and inflammation—that culminate in immune dysbiosis and systemic disease. Though exemplified here in the oral domain, this model may extend to other systemic diseases where paired immune engagements arise from distinct anatomical origins.

Experimental approaches—including murine models—are described to test the feasibility of this hypothesis. If validated, oral tolerance may provide a mechanistically grounded explanation for the co-emergence of chronic periodontitis and systemic chronic inflammation, thereby opening new perspectives or strategies for treating periodontitis-associated diseases.

## Data Availability

The original contributions presented in the study are included in the article/Supplementary Material, further inquiries can be directed to the corresponding author.
