# Supplementary material for: Oral tolerance, a potential driver of systemic disease risk in periodontal patients
Source: Front Dent Med. 2025 Dec 16;6:1700937. doi: 10.3389/fdmed.2025.1700937 (PMC12748255; doi:10.3389/fdmed.2025.1700937)
Supplement: Supplementary file 1 [file Table1.docx]

Supplementary Material

Table 2 Caption

Strains analyzed for attribution to Infective Endocarditis (IE) or Atherosclerotic Plaque (AP) are listed alphabetically. Binary attribution scores are shown in columns “IE” and “AP.” The combined attribution to Cardiovascular Disease (CVD), defined as IE + AP (where AP is only scored if IE score is 0), is listed in column “CVD.” The far left column shows the category into which the strain was sorted for display in Chart 1. The site of primary residence and GALT access assignment are shown along with additional characteristics of the strains. “Access via” – “S”, swallowing; “M”, mucociliary clearance; “P”, proximity; “n.a.”, not applicable. The typical “AGE” of colonization is listed as “i”, infant; “c”, childhood; “a”, adolescent to adult; “t”, transient; “e”, environmental. Gram Staining: “P”, positive; “N”, negative.

**Table 2. Strain List**

| ***SITE*** | **Strain** | **IE** | **AS** | **CVD** | **Site** | **GALT** | **Access via** | **AGE** | **Gram-Stain** | **Comments** |
| --- | --- | --- | --- | --- | --- | --- | --- | --- | --- | --- |
| **C** | ***Abiotrophia defectivea*** | 1 |  | 1 | Oral | GALT | S, P | i | P | Fac. anaerobe, under-detected |
| **A** | ***Acinetobacter baumannii*** | 1 |  | 1 | URT | GALT? | S, M | t | N | Strict Aerobe, Coccobacilli, |
| **C** | ***Actinomyces gerencseriae*** | 0 | 0 | 0 | Oral | GALT | S | e | P | Fac. anaerobe, filimentous rods |
| **C** | ***Actinomyces naeslundii*** | 0 | 0 | 1 | Oral | GALT | S | i | P | Anaerobe, microaerophile, pleomorphic rods |
| **C** | ***Actinomyces odontolyticus*** | 0 | 0 | 0 | Oral | GALT | S | e | P | Anaerobe / capnophilic, Rods, |
| **C** | ***A. actinomycetemcomitans*** | 1 |  | 1 | Oral | GALT | S | a | N | Fac. anaerobe, coccobacilli |
| **A** | ***Aminobacter aminovorans*** | 0 | 0 | 0 | Soil, GI | none | n.a. | a,t | N | Anaerobe |
| **E** | ***Bacillus cereus*** | 1 |  | 1 | GI | GALT | P | e | P | Fac.Anaerobe, Rods/spores/Motile |
| **B** | ***Bacillus subtilis*** | 0 | 0 | 0 | Skin, GI | No GALT | n.a. | t | P | Aerobic, rod, motile spore-forming |
| **A** | ***Borrelia burgdorferi*** | 0 | 0 | 0 | tick-borne | none | n.a. | t | N | Microaerophilic, spirochete, motile |
| **B** | ***Brevibacterium casei*** | 0 | 0 | 0 | Skin | no GALT | n.a. | t | P | Aerobe, rod |
| **A** | ***Brevundimonas diminuta*** | 0 | 0 | 0 | Enviro, Skin | None | n.a. | t | N | Aerobic, rod, motile, formerly *P. diminuta* |
| **A** | ***Burkholderia mallei*** | 0 | 0 | 0 | URT | GALT | M | t | N | Aerobe, Coccobacilli, , zoonotic exposure |
| **C** | ***Campylobacter rectus*** | 0 | 1 | 0 | Oral | GALT | S | i | N | Fac. anaerobe, curved rods, motile |
| **D** | ***Cardiobacterium hominis*** | 1 |  | 1 | Pharynx, URTl | GALT | S, M | c | N | Fac. anaerobe, rod, HACEK |
| **D** | ***Chlamydia pneumoniae*** | 0 | 1 | 1 | URT, LRT | GALT | M, S | c | N | aerobe, reticulate bodies, Intracellular |
| **E** | ***Citrobacter freundii*** | 0 | 0 | 0 | GI | GALT | P | i | N | Fac. anaerobe, Rods, flagella, motile, water, soil |
| **E** | ***Clostridium septicum*** | 0 | 0 | 0 | GI | GALT | P | e | P | Anaerobe, spores |
| **B** | ***Corynebacterium jeikeium*** | 1 |  | 1 | Skin | No GALT | n.a. | e | P | Aerobic, rod, |
| **D** | ***C. pseudodiphtheriticum*** | 0 | 0 | 0 | Pharynx | GALT | S, M | i,e | P | aerobe, , club shaped rods |
| **B** | ***Corynebacterium striatum*** | 1 |  | 1 | Nares, Skin | GALT | D, S | e | P | Fac. anaerobe, motile rod |
| **A** | ***Coxiella burnetii*** | 1 |  | 1 | Enviro aerosol | None | n.a. | n | N | Anaerobe, coccobacilli, intracellular |
| **B** | ***Cutibacterium acnes*** | 1 |  | 1 | Skin follicles | No GALT | n.a. | a | P | Anaerobe (aerotolerant), rods |
| **B** | ***Cutibacterium granulosum*** | 0 | 0 | 0 | Skin follicles | No GALT | n.a. | e | P | Anaerobe, rod, |
| **B** | ***Dermacoccus nishinomiyaensis*** | 0 | 0 | 0 | Skin | No GALT | n.a. | t | P | Aerobe, cocci, |
| **C** | ***Eikenella corrodens*** | 1 | 0 | 1 | Oral, URT | GALT | S, M | i | N | Fac. anaerobe, HACEK group |
| **E** | ***Enterobacter dissolvens*** | 0 | 0 | 0 | GI | GALT | P | i,e | N | Fac. anaerobe, Rods, motile |
| **E** | ***Enterobacter hormaechei*** | 0 | 1 | 1 | GI | GALT | P | e | N | Fac. anaerobe, Rods, motile |
| **E** | ***Enterococcus faecalis*** | 1 |  | 1 | GI | GALT | P | e | P | Fac. anaerobe, cocci |
| **E** | ***Enterococcus faecium*** | 1 |  | 1 | GI | GALT | P |  | P | Fac. anaerobe, cocci |
| **E** | ***Escherichia coli*** | 1 |  | 1 | GI, Oral, Pharynx | GALT | P, S | n | N | Fac. anaerobe, Rods |
| **C** | ***Filifactor alocis*** | 0 | 0 | 0 | Oral | GALT | S | a | P | anaerobe, rods |
| **C** | ***Fusobacterium nucleatum*** | 1 |  | 1 | Oral, Pharynx, Gut | GALT | S | i,e | N | anaerobe |
| **C** | ***Ganulicatella adiacens*** | 1 |  | 1 | Oral, urogenital | GALT | S | i | P | Fac. anaerobe, Cocci |
| **C** | ***Ganulicatella elegans*** | 1 |  | 1 | Oral | GALT | S | e | P | Fac. anaerobe, cocci |
| **C** | ***Gemella morbillorum**** | 1 |  | 1 | Oral, Pharynx | GALT | S, D | i,e | P | Fac. Anaerobe |
| **D** | ***Haemophilus influenzae*** | 1 |  | 1 | Pharynx, Sinus | GALT | D, M | i | N | Fac. Anaerobe |
| **D** | ***Haemophilus parainfluenzae*** | 1 |  | 1 | Pharynx | GALT | D, M | e | N | Fac. Anaerobe |
| **D** | ***Kingella denitrificans*** | 1 |  | 1 | Pharynx | GALT | M | e | N | Aerobe, HACEK |
| **D** | ***Kingella kingae*** | 1 |  | 1 | Pharynx, | GALT | S, D | e | N | Fac. Anaerobe |
| **E** | ***Klebsiella pneumoniae*** | 1 |  | 1 | Gut | GALT | P | i | N | Fac. anaerobe, Rods, encapsulated |
| **E** | ***Kocuria rosea*** | 1 |  | 1 | Salt Water, Skin | none | n.a. | a | P | Aerobe, cocci, |
| **A** | ***Legionella pneumophila*** | 1 |  | 1 | Water aerosol | none | n.a. | t | N | Aerobic, pleomorph .rod, motile. |
| **A** | ***Leuconostoc mesenteroides*** | 1 |  | 1 | Gut skin | none | n.a. | t | P | Fac. anaerobe, cocci |
| **E** | ***Listeria monocytogenes*** | 0 | 0 | 0 | GI | GALT | P | e | P | Fac. anaerobe, Fac. intracellular |
| **B** | ***Micrococcus luteus*** | 1 |  | 1 | Skin, URT | Confounding | n.a. | e | P | Aerobe, cocci, |
| **D** | ***Moraxella catarrhalis*** | 1 |  | 1 | Pharynx, | GALT | D, S | i,e | N | Aerobe, Diplococci, +-intracellular |
| **D** | ***Mycobacterium tuberculosis*** | 1 |  | 1 | URT, s | GALT | M , S | a | AFB | Aerobe, thin rods, =-. intracellular |
| **C** | ***Neisseria elongata*** | 1 |  | 1 | Oral | GALT | S | e | N | Aerobe, rods |
| **B** | ***Neisseria gonorrhoeae*** | 1 |  | 1 | Urogenital mucosa | No GALT | n.a. | n | N | Aerobic, diplococ, =- intracellulr |
| **D** | ***Neisseria meningitidis*** | 0 | 0 | 0 | Pharynx | GALT | D, M | a | N | Aerobe, diploccci |
| **B** | ***Neisseria sicca*** | 1 |  | 1 | Oral, Pharynx, URT | GALT | S, D | i,e | N | Aerobe, diploccci |
| **A** | ***Pantoea agglomerans*** | 0 | 0 | 0 | Puncture | none | n.a. | t | N | Fac. anaerobe, motile rod |
| **C** | ***Parvimonas micra*** | 1 |  | 1 | Oral | GALT | S | e | P | Anaerobe, cocci |
| **C** | ***Peptostreptococcus anaerobius*** | 0 | 0 | 0 | Oral, Skin, vagina | GALT | S | i,e | P | Anaerobe, cocci |
| **C** | ***Porphyromonas gingivalis*** | 0 | 1 | 1 | Oral | GALT | S | a | N | Anaerobe, rods |
| **C** | ***Prevotella buccae*** | 0 | 0 | 0 | Oral | GALT | S | e | N | Fac. anaerobe |
| **E** | ***Prevotella copri*** | 0 | 0 | 0 | GI, Oral | GALT | P | e | N | Anaerobe, rods, intracellr |
| **C** | ***Prevotella intermedia*** | 0 | 1 | 1 | Oral | GALT | S | c,a | N | Obl. anaerobe, short rods |
| **C** | ***Prevotella melaninogenica*** | 0 | 0 | 0 | Oral, URT | GALT | S, M | i | N | Anaerobe |
| **C** | ***Prevotella nigrescens*** | 0 | 1 | 1 | Oral | GALT | S | e | N | Aerobe, rod |
| **A** | ***Pseudomonas aeruginosa*** | 1 |  | 1 | Enviro, Ear, eye, URT | liminal zones | n.a. | t | N | Aerobe, rod, motile |
| **A** | ***Pseudomonas luteola*** | 0 | 0 | 0 | Puncture | none | n.a. | t | N | Aerobe, rod, motile puncture, contm.water |
| **C** | ***Rothia dentocariosa*** | 1 |  | 1 | Oral | GALT | S | i | P | Fac. anaerobe, Cocci |
| **C** | ***Rothia mucilaginosa*** | 1 |  | 1 | Oral, URT | GALT | S | i | P | Fac. anaerobe |
| **E** | ***Salmonella enterica*** | 1 |  | 1 | GI | transient GALT | P | t | N | Fac. anaerobe, Rods, motile, flagella |
| **B** | ***Staphylococcus aureus*** | 1 |  | 1 | Skin, Nasal | GALT | D | i | P | Facultative anaerobe, cocci |
| **B** | ***Staphylococcus epidermidis*** | 1 |  | 1 | Skin, liminial, GI | no GALT | n.a | e | P | Fac. anaerobe, cocci, |
| **B** | ***Staphylococcus haemolyticus*** | 1 |  | 1 | Skin, hosp | No GALT | n.a. | a | P | Fac. anaerobe, cocci, |
| **B** | ***Staphylococcus hominis*** | 1 |  | 1 | Skin | no GALT | n.a. | t | P | Fac. anaerobe, cocci, |
| **A** | ***Staphylococcus lugdunensis*** | 1 |  | 1 | Exogenous, Skin | no MALT | n.a. | t | P | Fac. anaerobe, cocci, |
| **B** | ***Staphylococcus pasteuri*** | 0 | 0 | 0 | Skin | no GALT | n.a. | a | P | Fac. anaerobe, cocci, |
| **E** | ***Streptococci agalactiae*** | 1 |  | 1 | GI, Pharynx | GALT | P | i | P | Fac. anaerobe, cocci |
| **E** | ***Streptococci dysgalactiae*** | 1 |  | 1 | GI, Skin, throat | GALT | P | e | P | Fac. anaerobe, cocci, (Group B strep) |
| **C** | ***Streptococcus anginous*** | 1 |  | 1 | Oral, GI | GALT | S | i | P | Fac. anaerobe, cocci |
| **E** | ***Streptococcus bovis*** | 1 |  | 1 | GI | GALT | P | c,a | P | Fac. anaerobe, cocci |
| **E** | ***Streptococcus constellatus*** | 1 |  | 1 | Oral, GI | GALT | S | e | P | Fac. anaerobe, cocci |
| **C** | ***Streptococcus cristatus*** | 0 | 0 | 0 | Oral, Pharynx | GALT | S | i,e | P | Fac. anaerobe |
| **C** | ***Streptococcus gordonii*** | 1 |  | 1 | Oral | GALT | S | i | P | Fac. anaerobe, cocci |
| **C** | ***Streptococcus mitis*** | 1 |  | 1 | Oral, Pharynx | GALT | S | i | P | Fac. anaerobe, mitis group |
| **C** | ***Streptococcus mutans*** | 1 |  | 1 | Oral | GALT | S | e | P | Fac. anaerobe, cocci |
| **C** | ***Streptococcus oralis*** | 1 |  | 1 | Oral, Pharynx | GALT | S | e | P | Fac. anaerobe, cocci |
| **C** | ***Streptococcus parasanguis*** | 1 |  | 1 | Oral | GALT | S | i | P | Fac. anaerobe, cocci |
| **D** | ***Streptococcus pneumoniae*** | 1 |  | 1 | Pharynx, Oral | GALT | M , D | i | P | Fac. anaerobe, mitis group |
| **D** | ***Streptococcus pyogenes*** | 1 |  | 1 | Pharynx | GALT | D, M | c | P | Fac. anaerobe |
| **C** | ***Streptococcus salivarius*** | 1 |  | 1 | Oral | GALT | S | i | P | Fac. anaerobe, cocci |
| **C** | ***Streptococcus sanguinis*** | 1 |  | 1 | Oral | GALT | S | i | P | Fac. anaerobe, cocci |
| **C** | ***Streptococcus sinensis*** | 0 | 0 | 0 | Oral | GALT | S | e | P | Fac. anaerobe, cocci |
| **E** | ***Streptococcus thermophilus*** | 0 | 0 | 0 | GI, dairy | GALT | P | t | P | Fac. anaerobe, cocci |
| **C** | ***Tannerella forsythia*** | 0 | 1 | 1 | Oral | GALT | S | a | N | Anaerobe, rod |
| **C** | ***Treponema denticola*** | 0 | 1 | 1 | Oral | GALT | S | a | N | Anaerobe, spirochete, motile |
